# Comment on: “Impact of Depth of Invasion in Node‐Negative Oral Tongue Cancer Treated With Surgery Alone”

**DOI:** 10.1002/kjm2.70170

**Published:** 2026-01-08

**Authors:** Erkan Topkan, Efsun Somay, Ugur Selek

**Affiliations:** ^1^ Department of Radiation Oncology, Faculty of Medicine Baskent University Adana Turkey; ^2^ Department of Oral and Maxillofacial Surgery, Faculty of Dentistry Baskent University Ankara Turkey; ^3^ Department of Radiation Oncology School of Medicine, Koc University Istanbul Turkey

Dear Editor,

We extend our congratulations to Hsiao et al. for their study evaluating the prognostic significance of depth of invasion (DOI) in node‐negative oral tongue squamous cell carcinoma (OTSCC) treated exclusively with surgery [1]. Given the ongoing debate on how best to incorporate DOI into prognostication and clinical decision‐making, the authors deserve recognition for assembling a relatively homogeneous cohort of 243 patients and for demonstrating the independent prognostic role of DOI across various survival endpoints. However, we would like to highlight several methodological and interpretive considerations that may limit the generalizability of their findings.

First, the authors made efforts to establish uniformity in their study by excluding patients with nodal positivity, those receiving adjuvant therapy, and cases with close surgical margins. Nevertheless, this strategy is prone to introducing unintentional selection bias, leading to a cohort that is highly favorable and characterized by relatively low event rates. Indeed, the reported 5‐year overall survival rate exceeded 87%, a figure markedly higher than those found in many comparable clinical series encompassing a wider range of patient characteristics and treatment settings [2]. Unfortunately, such inflation of positive outcomes may restrict the extrapolation of these results to a broader OTSCC population, particularly for patients treated outside high‐volume tertiary centers, where treatment protocols and patient demographics can vary significantly.

Second, while the authors successfully developed a DOI‐based nomogram that demonstrates reasonable concordance with a C‐index of 0.700, surpassing the AJCC TNM staging system's C‐index of 0.618. However, the lack of external validation significantly limits the immediate clinical applicability of this nomogram. As highlighted in recent comprehensive reviews, nomograms necessitate thorough calibration and validation across independent cohorts to ensure their reliability and effectiveness before they can be widely adopted in clinical practice [3]. Additionally, critical histopathological features known to correlate with DOI, such as tumor budding, which indicates aggressive cancer behavior, and the worst pattern of invasion, which reflects the most aggressive tumor growth pattern, were not incorporated into the nomogram [4]. Therefore, to strengthen the evidence base, it would be beneficial to conduct prospective multi‐institutional studies or to analyze data from national cancer registries, as these approaches would provide a more rigorous assessment of the nomogram's performance across diverse patient populations. At the same time, the inclusion of these features could further enhance the predictive accuracy of the model and improve risk stratification beyond the insights gained from DOI alone [2].

And third, the authors reported that neck dissection mitigated the negative prognostic impact of DOI on regional recurrences. However, the analysis did not adequately address potential confounding by indication, as patients selected for elective neck dissection may have differed systematically from those treated with observation in ways that were not fully represented by the recorded variables. In the absence of propensity score adjustment or multivariable modeling incorporating surgical decision factors, the observed protective effect of elective neck dissection should be interpreted with caution before changing current oncological practice routines in these groups of patients, considering the potential futile complications of the procedure [5].

## Conflicts of Interest

The authors declare no conflicts of interest.

## Data Availability

Data sharing not applicable to this article as no datasets were generated or analysed during the current study.
